# Decreased morbidity following long saphenous vein harvesting using a minimally invasive technique: a randomised controlled trial comparing two techniques for long saphenous vein harvest

**DOI:** 10.1186/1749-8090-1-15

**Published:** 2006-06-07

**Authors:** Zahid Mahmood, Sammy Al Benna, Udim Nkere, Andrew Murday

**Affiliations:** 1 Department of Cardiothoracic Surgery, Glasgow Royal Infirmary, Glasgow, UK

## Abstract

**Objectives:**

The objective of this study was to compare the morbidity associated with long saphenous vein harvesting using the traditional open technique (A) against a minimally invasive technique using the Mayo vein stripper (B) that involves multiple short incisions.

**Design:**

We conducted a prospective randomized controlled study in 80 patients undergoing first time coronary artery bypass grafting. Pain and healing was assessed on each postoperative day. Rings of long saphenous vein were subjected to organ-bath evaluation of endothelium-dependent and endothelium-independent relaxation.

**Results:**

Three patients were excluded from the study, leaving 38 patients in Group A and 39 in Group B. With respect to operative procedure, Group A had a greater length of vein harvested than Group B. There was no statistical difference in pain scores and endothelium-dependent or endothelium-independent relaxation between the two groups. However there were significantly more infections in Group A compared with Group B.

**Conclusion:**

Harvesting vein through multiple incisions using the Mayo vein stripper is quicker, results in fewer infections and has no deleterious effect on endothelial function compared to open technique.

## Background

The traditional method for harvesting this vein involves the use of a long incision from ankle to groin. A frequent observation after coronary artery bypass graft surgery is that many patients complain more about their leg wound than they do about their median sternotomy. The long incision is associated with significant intra-operative and postoperative complications [1]. Impaired leg wound healing has been reported to occur in up to 25% of patients [2]. Wound complications including infections, hematoma formation, cellulitis and saphenous neuropathy prolong recovery [3].

The use of minimal invasive saphenous vein harvesting has been advocated in an effort to minimize such wound related problems [4-6]. There is already some evidence that these methods may reduce leg wound complications [6], although they have not yet gained widespread use.

Several techniques are available for minimal invasive vein harvesting, but all necessitate traction on the vein to maximize surgical visibility and enable side branch ligation. Excessive surgical manipulation of saphenous vein impairs endothelial cell function and reduces the bioavailability of nitric oxide [7-9]. This endothelial injury promotes platelet and leukocyte adhesion that in turn can result in smooth muscle cell proliferation that exacerbates the intimal hyperplasia that is a common cause of vein graft occlusion [10-12]. Thus, functional integrity of the harvested and prepared saphenous vein has important implications for immediate and long-term graft patency

## Materials and methods

### The patients

From May 2002 to May 2003, 80 patients scheduled for elective first time coronary artery bypass grafting were recruited into the study. Study participants had isolated coronary artery disease that required at least part of their revascularization to be done using the LSV. Exclusion criteria included patients undergoing emergency coronary artery bypass grafting and those with severe varicose veins. The study was approved by the Local Research and Ethics Board at Glasgow Royal Infirmary. Before enrollment and randomization each participant provided written informed consent.

### Surgical techniques

Two consultant cardiac surgeons (UN and AM) carried out all of the operative procedures reported in this study.

#### Long incision (open) saphenous vein harvesting (Group A)

The incision was commenced just above the medial malleolus. The vein was identified and cleared of all adventitia and connective tissue using sharp and blunt dissection. The skin was incised over the whole length of the vein to the required length and careful dissection was used to isolate the vein in situ, with attention given to avoid unnecessary trauma to the vein or its tributaries. Side branches were ligated with 4/0 ethibond ligatures on the vein side and metal clips on the patient side. The leg wound was closed in layers and a full length pressure dressing was applied.

#### Minimal invasive technique for long saphenous vein harvest (Group B)

A 2 cm longitudinal incision was made above the medial malleolus. The long saphenous vein was identified and cleared of all adventitial and connective tissue using sharp dissection. The distal end of vein was tied. The proximal part was also tied and about 6–8 cms of thread was left with that end. The vein was then divided between the two tied ends. The end with a thread was passed through the ring in the Mayo vein stripper and forward pressure was applied to the vein stripper in the direction of usual vein anatomy while applying traction on the vein through the length of thread. Whenever resistance was felt on the vein stripper, a small incision (2 to 3 cm) at the area where resistance was felt was made. With a combination of sharp and blunt dissection any branch of the saphenous vein at that site was isolated and ligated using a 4/0 ethibond ligature on the vein side and a metal clip on the patient side. The same process was repeated with multiple short incisions until the required length of vein was obtained. The skin incisions were closed with skin staples and full-length pressure dressing applied.

In common to both techniques of harvest, the vein was inflated with heparinised blood to check for any unidentified side branches or tears in the vein. Any that were identified were either ligated with a 4/0 ethibond suture or closed with a 6-0 prolene suture.

### Randomization

Patients were randomized immediately prior to surgery by minimization using a computer program [13]. Minimization has the advantage that differences in important patient variables that might otherwise occur by chance can be avoided. The following patient characteristics were employed for minimization: diabetes, peripheral vascular disease, age and gender.

### Wound complications

Wound complications were assessed using the ASEPSIS [14] scoring method (Table 1). All incisions were carefully assessed by a single observer on each day of the in-patient stay. The ASEPSIS score measures erythema, exudates and wound separation. A numerical score is calculated according to the proportion of the wound affected by each of these characteristics (table 5). All discharging wound were swabbed and any bacterial growth identified by standard methods. Since some patients were discharged by the 5th postoperative day we calculated the sum of the first five daily scores for each patient.

### Pain measurement

A visual analogue score was used to assess postoperative leg pain [15,16]. This consisted of a 10 cm long straight line with extreme limits marked with perpendicular lines and appropriate labels, but with no words or numbers between the endpoints. The patient was asked to mark the line with a cross at a point that represented their current level of pain. The sensitivity of this type of pain evaluation has been previously validated [15]. Pain assessment was carried out daily until hospital discharge.

### Vasomotor studies

Of the 79 patients in the study 29 were evaluated for vasomotor studies, 14 from Group A and 15 from Group B. A saphenous vein segment was removed prior to distension and immediately rinsed without pressure, immersed in iced oxygenated Krebs-Henseleit buffer [17] and transported to the laboratory. These vein segments were divided into three or four rings approximately 4 mm long that were mounted in a 25 ml organ bath. The tension was recorded directly onto a computer. Optimal resting tension was determined in baseline studies. Rings were then pre-contracted with phenylephrine (30 mmol/l). Endothelium-dependent relaxation was evaluated by cumulative addition of calcium ionophore A23187 (0.1 to 10 mmol/l) and endothelium-independent relaxation was evaluated by sodium nitroprusside (0.001 to 0.1 mmol/l) [18]. Relaxation to the NAD (P) H oxidase inhibitor apocynin (10 to 1000 mmol/l) was studied to assess the contribution of super oxide to vasoconstriction.

### Statistics

Statistical analysis were performed using Student's t-test to compare parametrically distributed variables, the Mann Whitney test to compare non-parametrically distributed variables and the Chi-squared to compare discrete variables.

## Results

Of eighty patients undergoing coronary artery surgery in the study period, one patient was unavoidably excluded from the study before vein harvest commenced. Two patients who died within the first 48 hours were excluded from further analysis, leaving 38 patients in group A and 39 in group B. There were no differences in the demography of the patients between the two groups (Table 2). No patient from group B was converted to the traditional open technique.

**Table 1a:** The ASEPSIS wound score

Criterion	Description	Points
**A **Additional treatment	Antibiotics	10
	Drainage of pus under local anesthetics	5
	Debridement of wound (General anesthetics)	10
**S **Serous discharge	Daily	0–5
**E **Erythema	Daily	0–5
**P **Purulent exudates	Daily	0–10
**S **Separation of deep tissues	Daily	0–10
**I **Isolation of bacteria		10
**S **Stay in hospital prolonged over 14 days		5

**Table 1b:** Points scale for the daily wound inspection

Wound characteristic	Proportion of wound affected (%)					
	
	0%	<20%	20–39%	40–59%	60–79%	>80%
Serous exudates	0	1	2	3	4	5
Erythema	0	1	2	3	4	5
Purulent exudates	0	2	4	6	8	10
Separation of deep tissues	0	2	4	6	8	10

**Table 1c:** The total score with the category of infection

Total score	Category of infection
0–10	Satisfactory healing
11–20	Disturbance of healing
21–30	Minor wound infection
31–40	Moderate wound infection
>40	Severe wound infection

**Table 2:** Preoperative demographics

	Group A	Group B
Age (years: mean ± 1sd)	63 (8)	65 (8)
Male sex	29	29
NYHA class (I/II/III/IV)	0/8/14/15	0/8/15/16
Smoker	14	14
Diabetic		
Type 1	3	2
Type 2	6	8
Obese (BMI>30)	10	8
Peripheral vascular disease	9	9
Venous stasis disease	5	9

**Table 3:** Intraoperative data

	Group A	Group B	
CPB time (mins: mean ± 1sd)	85 (23)	78 (15)	ns
Theatre time (mins: mean ± 1sd)	200 (32)	192 (24)	ns
Vein harvest site:			
Calf only	35	35	
Calf and thigh	3	4	
Total no of grafts (mean ± 1sd)	3.3 (0.7)	3.2 (0.5)	ns
No of vein grafts (mean ± 1sd)	1.9 (0.7)	1.6 (0.5)	ns
Length of vein harvested (cm: mean ± 1sd)	31 (13)	25 (9)	p = 0.018
Length of wound (cm: mean ± 1sd)	35 (13)	16 (6)	p < 0.001
Number of incisions (mean ± 1sd)	1.2 (0.4)	6.9 (2.7)	p < 0.001
Vein harvest time (mins: median + IQR)			
Stage 1	16 (10)	11 (14)	p = 0.01
Stage 2	9 (6)	2 (1)	p < 0.001
Stage 3	2 (2)	2 (4)	p = 0.5
Total	26 (16)	15 (22)	p = 0.002

### Intra operative findings

The total operative time taken to harvest vein was divided into 3 parts; stage 1: start of skin incision to removal of vein, stage 2: incision closure and stage 3: vein preparation. Adding the 3 gives the total vein operation time (Table 3). Technique B was significantly quicker whilst requiring no increase in the time required to prepare the vein. Time was saved both during removal of the vein and during skin closure. There was no statistical difference in length of vein harvested but total wound length and number of incisions was less in Group B (Table 3).

### Postoperative pain

Although the analogue scale pain scores were less in Group B than in Group A, the differences did not reach statistical significance either for maximum pain score or total pain score over the 5 first postoperative days. (Table 4)

**Table 4:** Pain scores in first 5 post operative days

	Group A	Group B	Student' s t-test
Maximum pain score (mean ± 1sd)	4.4 (2.0)	3.8 (2.1)	P = 0.17
Total pain score (mean ± 1sd)	10.2 (5.5)	9.1 (5.8)	P = 0.43

**Table 5:** Relaxation of long saphenous vein rings in response to calcium ionophore, sodium nitroprusside and apocynin.

Agonist	Group A	Group B	
Apocynin (mean ± 1 sem)	22.3 (8.7)	20.6 (8.6)	p = 0.7
Calcium Ionophore (mean ± 1 sem)	18.4 (7.0)	17.6 (7.5)	p = 0.4
Sodium Nitroprusside (mean ± 1 sem)	37.6 (12.1)	40.1 (13.1)	p = 0.6

### Infection and healing

**Figure 1:**
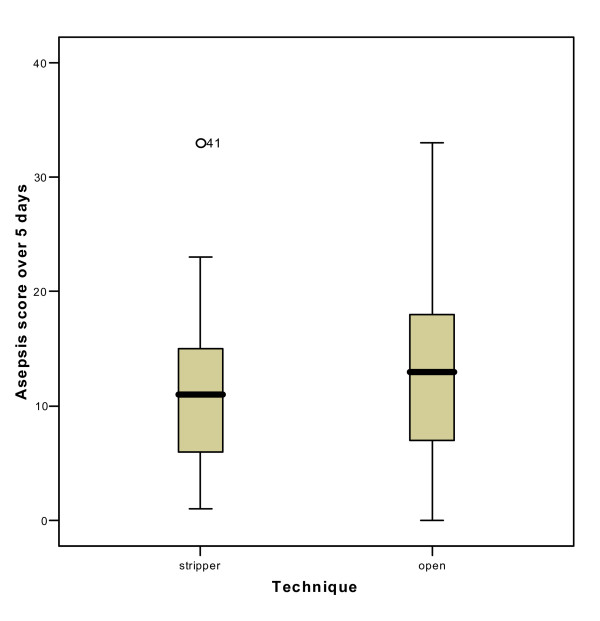


The accumulated ASEPSIS scores for the first five postoperative days are shown in Figure 1. When the scores are categorized according to the grades given in Table 1c, there were 8 patients in Group A with a minor wound infection or worse, compared to only 2 in Group B. This reaches statistical significance using a Chi-squared test (p < 0.001).

### Vasomotor studies

Long saphenous vein rings harvested from Group A and Group B showed similar relaxation to calcium ionophore, sodium nitroprusside and apocynin. (Table 5)

## Discussion

Modern management of CABG surgery patients emphasizes an early return to normal activities. In this regard early mobilization after surgery plays an important part in the process of recovery. In turn, any reduction in morbidity from the saphenous vein harvest procedure will promote early mobilization and speed rehabilitation.

In this randomized study we found that compared to the traditional open method of harvesting long saphenous vein, a less invasive technique employing a Mayo vein stripper was quicker and provided significantly improved wound healing. There are other instruments available but the Mayo stripper has the advantage of being reusable and is therefore less expensive than disposable devices.

Although we only examined endothelial function in a sample of the harvested veins, we found no evidence that the manipulation of the vein, consequent upon using the Mayo stripper, affected endothelial function any more than the traditional open harvest method. In order to eliminate the effect of surgical expertise, we used 2 very experienced surgeons who were equally comfortable with either technique to carry out all of the vein harvesting. However, we see no reason why the less invasive technique cannot be taught to surgical trainees and surgical assistants, or why they should not be able to carry it out to an equally high standard.

We failed to demonstrate any significant difference in the perception of pain from the leg wounds. In view of the fact that there is a significant difference in the overall length of the skin incisions between the two patient groups this is a little surprising, and does not reflect our subjective observations.

In conclusion, this study supports the notion that harvesting vein through multiple incisions using the Mayo vein stripper is quicker, results in better wound healing and has no deleterious effect on endothelial function compared to the open technique.
